# Human errors in emergency medical services: a qualitative analysis of contributing factors

**DOI:** 10.1186/s13049-024-01253-7

**Published:** 2024-08-30

**Authors:** Anna Poranen, Anne Kouvonen, Hilla Nordquist

**Affiliations:** 1 Faculty of Medicine, University of Helsinki, Helsinki, 00014 Finland; 2 Faculty of Social Sciences, University of Helsinki, Helsinki, 00014 Finland; 3 Centre for Public Health, Queen’s University Belfast, Belfast, BT12 6BA Northern Ireland; 4 South-Eastern Finland University of Applied Sciences, Kotka, 48220 Finland

**Keywords:** Emergency medical services, Patient safety, Human error, Paramedic

## Abstract

**Background:**

The dynamic and challenging work environment of the prehospital emergency care settings creates many challenges for paramedics. Previous studies have examined adverse events and patient safety activities, but studies focusing on paramedics’ perspectives of factors contributing to human error are lacking. In this study, we investigated paramedics’ opinions of the factors contributing to human errors.

**Method:**

Data was collected through semi-structured individual interviews (*n* = 15) with paramedics and emergency medical field supervisors in Finland. The data was analyzed using inductive content analysis. Consolidated criteria for reporting qualitative research were used.

**Results:**

Contributing factors to human errors were divided into three main categories. The first main category, *Changing work environment*, consisted of two generic categories: *The nature of the work* and *Factors linked to missions*. The second main category, *Organization of work*, was divided into three generic categories: *Inadequate care guidelines*,* Interaction challenges* and *Challenges related to technological systems*. The third main category, *Paramedics themselves*, consisted of four generic categories: *Issues that complicate cognitive processing*, *Individual strains and needs*,* Attitude problems* and *Impact of work experience*.

**Conclusion:**

Various factors contributing to human errors in emergency medical services (EMS) settings were identified. Although many of them were related to individual factors or to the paramedics themselves, system-level factors were also found to affect paramedics’ work and may therefore negatively impact patient safety. The findings provide insights for organizations to use this knowledge proactively to develop their procedures and to improve patient safety.

**Supplementary Information:**

The online version contains supplementary material available at 10.1186/s13049-024-01253-7.

## Background

Healthcare is considered a high-risk industry similarly to aviation industry where human error management has been acknowledged already for decades [1]. Healthcare systems worldwide have learned safety procedures from other safety critical organizations, and a great deal of attention has been focused on eliminating human errors and improving patient safety. In complex and changing health care work environments, many factors contribute to errors, not all of which can be eliminated [1, 2]. Human action is valuable and necessary because it withstands variability and fine adjustment that is needed in dynamic and complex systems [3, 4]. The variability of human action can lead to both successes and failures. Therefore, contributing factors which lead to variations of human actions, and sometimes undesirable outcomes should be identified because safety is not improved by simply eliminating errors [3]. However, if patient safety protocols are deviated from, organizations should not only investigate errors related to human behavior, but they should also explore how interactions between the system and the individuals may have failed [4]. Human error is not a cause of adverse events [4]. Human error can be defined as a situation where performance variability is needed, and the outcome is undesirable in the end. Secondly, under normal circumstances, the action leads to a desirable outcome [4]. In contrast, unfamiliar work circumstances or a distraction causes a loss of focus that leads to an error [5].

Emergency medical services (EMS) work environment is dynamic and challenging, and the risk for errors is high. Previous studies have indicated that fatigue and shift work can increase the risk of medical errors and negatively impact patient safety [6, 7]. Critically ill patients and organizational factors, such as a deviation of standard of care or insufficient training can also create a risk for adverse events [8]. Furthermore, difficulties related to decision-making can affect patient safety [8, 9].

In the EMS setting, human errors have been studied since the 1980s and, a proactive approach is recommended for exploring factors that affect errors [10, 11]. Previous studies have investigated medication errors, patient safety activities and adverse events in the prehospital emergency care setting [8, 11–13], but little is known about factors contributing to human errors from paramedics’ perspectives. Proactive exploration of contributing factors to errors can provide new research understanding of this area and improve patient safety in the EMS setting. Therefore, the research question for this study was as follows: In paramedics’ opinions, what kinds of issues contribute to human error?

## Materials and methods

### Design

A qualitative study with semi-structured interviews and inductive content analysis was implemented to investigate human errors from paramedics’ perspective, capturing their lived experiences and views of this complex issue [14]. The consolidated criteria for reporting qualitative research (COREQ) checklist [15] were used for reporting this study and are outlined in Additional file 1.

### Setting

This study was carried out in Finland in 2020. At that time 21 hospital districts organized EMS in their areas. The hospital districts could provide EMS by themselves, in cooperation with local rescue services or by outsourcing the services to the private sector. In one hospital district, there could be more than one EMS provider organization. All EMS organizations are guided by the Ministry of Social Affairs and Health and national legislation [16, 17]. The Finnish EMS consists of advanced-level EMS units (staffed with at least one paramedic and a practical/registered nurse or a firefighter) and basic-level EMS units (staffed with one healthcare professional, e.g., a practical nurse who has specialized in prehospital emergency care and another practical/registered nurse or a firefighter) [17]. In Finland, advanced-level paramedics are either registered nurses with at least three-and-a-half years of training in a University of Applied Sciences (UAS) and additional prehospital emergency care specialization, or emergency care nurses with at least four years of training in UAS. Each hospital district had at least one EMS field supervisor who was responsible for the operational aspects of EMS. EMS field supervisors are advanced-level paramedics with sufficient work experience and operative leadership training and they were operating by their own units [17]. In addition, each hospital district had at least one on-call EMS physician who could always be requested for care instructions by Finnish paramedics. Finland also has helicopter EMS units, and in some districts, EMS physicians operate their own ground-units as well [17, 18].

### Participants

This study focused on paramedics and EMS field supervisors working in the EMS setting. The inclusion criteria were: (1) advanced-level or basic-level paramedic or EMS field supervisor with any length of work experience and (2) at the time of the study, worked in EMS. The convenience sampling method was used which is common in qualitative studies such as ours [19]. Participants were recruited via social media; in June 2020, a recruitment ad was posted in the Finnish Facebook group *Ensihoidon uutiset* (“News of Prehospital Emergency Medical Services”), which at the time had over 5,000 members working in EMS settings across Finland. Potential participants were asked to contact the first author via Facebook Messenger to receive more information about the study, after which, they confirmed their participation via email.

Eighteen people initially contacted the first author. Of these, two did not confirm their participation, and one wanted a different method of data collection. In total, 15 people confirmed their participation in the study. These participants were advanced-level paramedics and EMS field supervisors (later, paramedics); nine women and six men from seven EMS organizations in Finland, which represented eastern, western, northern, and central parts of Finland.

### Data collection

To enable a dialogue between the interviewer and the participant, semi-structured individual interviews were used to collect the data [14]. The interview guide, which addressed the knowledge gaps in the literature, was formulated by the first and the last authors. An external expert on system safety and human factors was asked to assess the appropriateness of the interview guide, after which a pilot interview was conducted with a potential study participant. A few changes were made based on the expert’s comments and the pilot interview.

The first author, an advanced-level paramedic with several years of experience in EMS, conducted the interviews. During the interviews, open and trusting dialogues were maintained to ensure that the interviewer did not influence the participants’ responses.

The interviews began with the question, “What does human error mean to you?” Subsequent questions encouraged the participants to describe the issues and situations, they believed to be linked to or affect human error in EMS settings. While the interview guide was predesigned, most of the follow-up questions were formulated based on the participants’ earlier responses and the interviewer’s notes that were written during the interviews. For instance, a follow up question was, “You said a long work shift can create a risk for errors, how will this affect working in the EMS in your opinion?” The interview guide is displayed in Additional file 2. Each interview was audio recorded and lasted between 40 and 75 min. Interviews were carried out in person (*n* = 8), online (*n* = 4), and by phone (*n* = 3), between July and October 2020. After conducting 13 interviews, the responses began to repeat themselves, yet interviews were conducted with all the paramedics who volunteered for the study.

The audio recordings were transcribed verbatim by the first author. All the interviews were assigned numerical codes and pseudonymized, and no personal information was included. Confidentiality of identity was guaranteed throughout the study process.

### Data analysis

The paramedics’ opinions on factors contributing to human errors were analyzed using inductive content analysis and the process followed the phases described by Elo and Kyngäs [20]. The first author read the transcripts carefully several times to obtain an overall understanding of the data. Then, short sentences were chosen as units of meaning, and the coding began. The contents answering to the research question were marked in the text, and the headings describing all aspects of the content were written in the text while it was being read. This was done by the first author without the use of any analysis software. To ensure the trustworthiness of the coding process and the correctness of the interpretation of participants’ responses, the first and the last author discussed and reviewed the process together. The last author has several years of both academic research experience and supervisory experience in the EMS setting, enabling a comprehensive understanding of the research method.

The headings were collected into a chart, and overlaps were removed. Then grouping began and similar content belonging together were grouped into subcategories and named using content-characteristic words. Then, the similar and related subcategories were grouped into broader generic categories and named. Finally, the main categories were formed based on the related generic categories [20]. An example of category grouping is shown in Additional file 3. The first and the last author worked together to group the categories. During the process, to ensure the trustworthiness of this study, the categories were reviewed and compared against the original data several times, and the main categories were formulated after profound reflection. The first, second, and last authors collaborated to finalize the categories. The second author has extensive experience in academic research, methodology, and supervision, as well as a broad understanding of occupational health and related social phenomena.

## Results

**Fig. 1:**
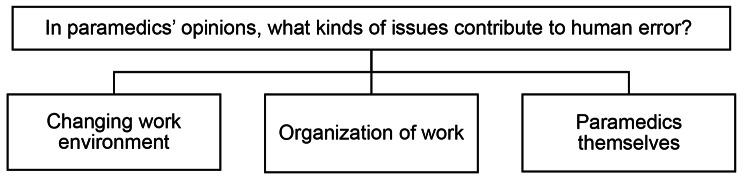
Factors contributing to human errors in EMS, according to paramedics

Three main categories were formed: (1) *Changing work environment*, (2) *Organization of work*, and (3) *Paramedics themselves*. An overview of the whole analysis is displayed in Additional file 4. Figure 1 provides an overview of the main categories.

### Changing work environment

**Fig. 2:**
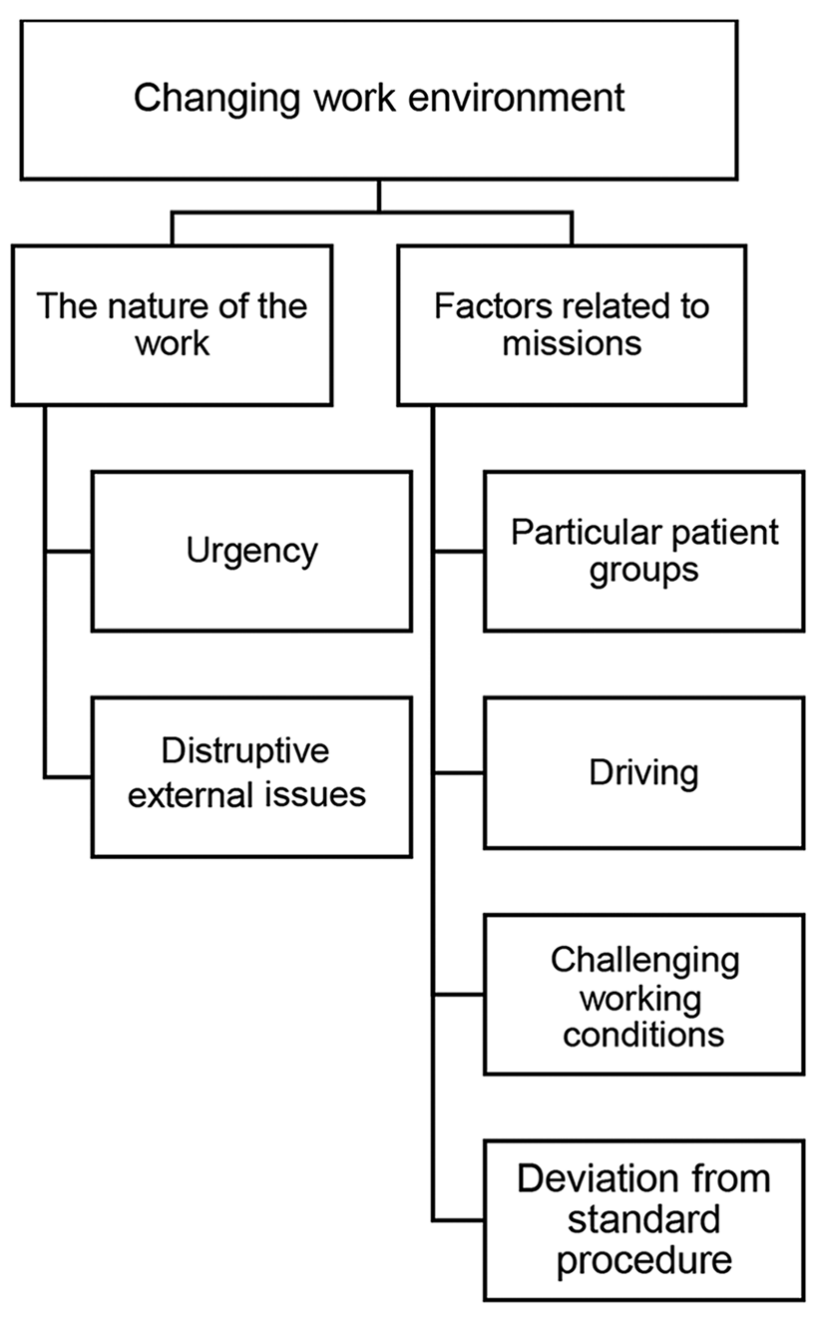
An overview of the first main category, *Changing work environment*

The main category *Changing work environment* consisted of two generic categories: *The nature of the work* and *Factors related to missions*. These categories describe aspects that are related to the unique work environment of the EMS. An overview of the first main category can be seen in Fig. 2.

#### The nature of the work

***Urgency.*** According to the participants, urgency is a factor that contributes to human errors. In their perspectives, a sense of urgency adds pressure, which in turn may lead to carelessness, and, subsequently, to errors. Participants explained that emergency missions can lessen one’s situational awareness and vigilance and therefore one’s control, which may cause errors to occur. Moreover, many missions include the added pressure of needing to be managed quickly so that an ambulance will be available for the next mission. Field supervisors may also cause a sense of urgency if they oversee how long paramedics stay at the scene.

*There is a rushed situation*,* and you are like*,* “Okay*,* it is this*,*” and then you give the wrong dose [of medication to the patient] — that rushed situation has created pressure in a way*,* and that is why an error happens. (P12)*

***Disruptive external issues.*** Based on the interviews, external factors can lead to human errors because they disturb the paramedics’ work and can distract them. A paramedic may not understand or notice something relevant, resulting in an error. For example, if a patient’s family members disagree with or argue with the paramedics, it can complicate the situation and make the paramedics’ work difficult, as it requires the paramedics’ attention to be diverted from the patient. According to the participants, families and other bystanders must be taken into account but if they feel that they are not noticed, a mutual agreement of the patient’s care can disappear and a risk for error can occur. Radio communication and ear buds can also be distracting.

*Of course*,* you must wear ear buds all the time and you have to listen to certain channels*,* and if you are focusing on something… it will disturb your own work. (P3)*

#### Factors related to missions

***Particular patient groups.*** The participants described particular patient groups that can affect work circumstances and may therefore contribute to errors.

Based on the interviews, the presence of critical illness can make circumstances challenging and these acute emergency situations can cause a sense of urgency and time pressure for paramedics, which can lead to forgetting something, such as when a paramedic must administer the urgently needed medication quickly. According to the opinions of the participants, these kinds of circumstances may contribute to errors. Critical procedures can cause pressure that can worsen the paramedics’ concentration in the missions and control of the situation as a whole can be lost. In addition, acute and mentally demanding missions and unstable patients requiring immediate care can cause errors because of the emergency. Moreover, a paramedic’s work may be affected by missions with similar critically ill patients because they may not remember which patient needs which medication or dose.

*We had a patient with an epileptic seizure the previous day*,* and I had given them an intranasal medication. Without thinking*,* I gave the same dose of medication intravenously to the next patient*,* like I had a day before. (P15)*

The participants noted that the factors related to frequent callers may also expose paramedics to errors, as several visits to the same address can increase paramedics’ disregard, and something might go unnoticed during these missions. This disregard may manifest as not paying enough attention. Patients with substance abuse problems, especially patients with alcohol problems, were mentioned as a particular group of frequent callers. In such cases, a patient may be left at home after an incomplete assessment. Furthermore, verbally difficult patients who argue against or otherwise do not cooperate with the paramedics can make the paramedics’ work more complicated and divert attention to potentially irrelevant things. This may contribute to errors.

*That disregard in terms of particular missions and patient groups—you don’t have enough strength with these same situations. We visit them 10 times for the same reason*,* and one day there is a real symptom*,* but you don’t have an interest in this patient anymore and when there is a real symptom*,* paramedics may ignore that because of the patient’s background. (P8)*

***Driving.*** Emergency response driving was seen as a stress factor per se; when paramedics drive quickly with lights and sirens on, it can lead to them making errors. A lack of communication between working pairs during emergency response driving was also mentioned in the interviews. Furthermore, participants said that falling asleep while driving and lack of local knowledge were additional factors that contribute to errors.

*When you switch on the lights and sirens, they are already stress factors. (P11)*

***Challenging working conditions.*** The participants stated that a forest, for example, or a container can create challenging working conditions. These environments can influence the management of a mission because they are unexpected. Unpleasant scenes or health and safety risks can cause paramedics to hurry to leave the scene of the mission and thus may contribute to human error.

*Challenging circumstances… that place that is absolutely not normal*,* or somewhere high up*,* somewhere where you must be suddenly able to work [to take care of the patient]. (P9)*

***Deviation from standard procedure.*** According to the participants, paramedics may provide incorrect care if they rely too heavily on the mission code, and the patient assessment is not done systematically and thoroughly. According to the participants, too many EMS workers involved in a situation can result in unintended carelessness where individuals are not working together, which in turn can lead to unstructured care. This can impact patient safety and was mentioned as a contributing factor to human error.

*We start to give medication and there is a crowd… somebody pulls the medicine into the syringe and says*,* “I have done it.” Another next to them watches it happen but doesn’t look carefully*,* and then there are two milligrams instead of one*,* or something like that. That circumstance makes the situation… a lot of people*,* a little bit of pressure. (P12)*

According to the participants, not double-checking the medications can contribute to human error. For instance, if the partners work too well together, they may decide that they do not need to double-check the medication, or it may be forgotten.

*A classic mistake — you didn’t remember to double-check the medication*,* did not remember or did not bother to do that*,* and then an error happens. (P3)*

Rarely given treatments were also described as contributing to errors, as something essential may not be noticed. In addition, new and rarely used medications and inexperience with them can cause mental pressure. The participants said that if a paramedic is not competent in using a medication or does not trust themselves or the medication itself, errors may occur.

*Something may not be noticed when the stress is increasing so much and if you haven’t been or have rarely been on a so-called tough mission. (P9)*

### Organization of work

**Fig. 3:**
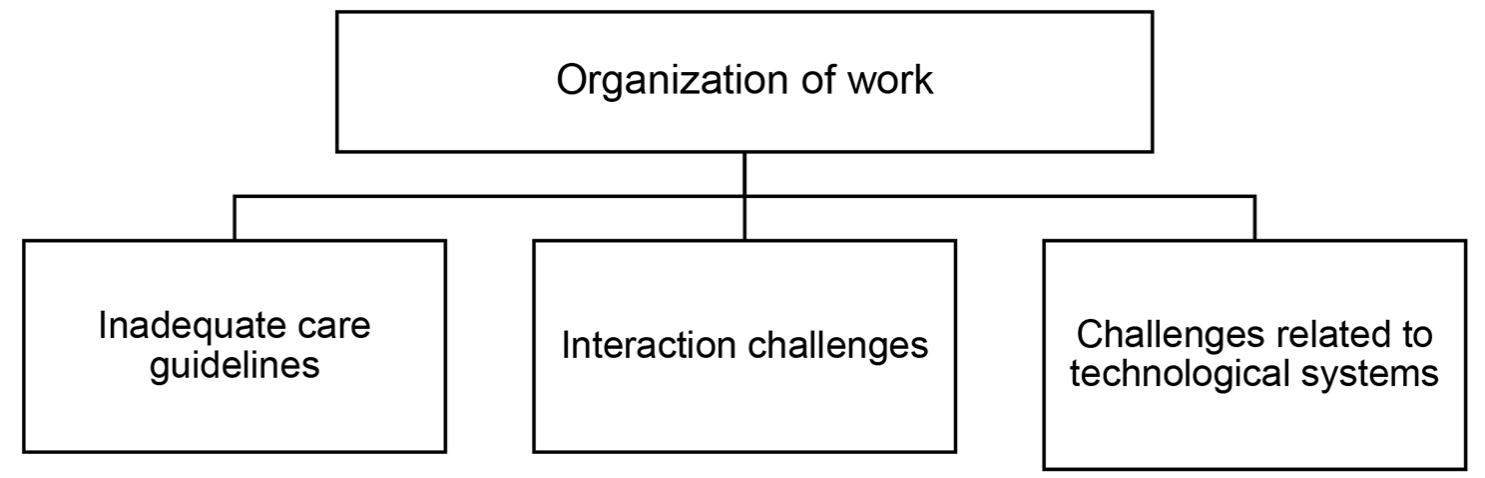
The second main category *Organization of work*

The category *Organization of work* consisted of three generic categories: *Inadequate care guidelines*, *Interaction challenges* and *Challenges related to technological systems*. These categories are formed with aspects that are mostly related to the organizational level. An overview of the second main category is presented in Fig. 3.

#### Inadequate care guidelines

According to the participants, if a standard operating procedure (SOP) is unclear, a paramedic may choose the wrong treatment. A lack of SOPs was also mentioned as leading to unsystematic and unstructured actions. The participants felt that concrete SOPs are needed because something essential may be forgotten even if they have practiced any special treatments or procedures beforehand. Paramedics may not know or be unsure about how to manage missions, procedures, and treatments if they do not have sufficient SOPs. Participants said that they sometimes have to make difficult decisions in unclear situations if they do not have adequate SOPs, and these decisions may be wrong for a patient. Moreover, recent updates to SOPs can also contribute to errors.

*Guidelines that are poorly made—that is why a paramedic might understand the guideline wrong [and an error may happen]. (P13)*

Based on the interviews, working in the border of two EMS operating areas and different options for follow-up care in neighbouring areas can make it challenging for paramedics to remember the care alternatives available in both their own and other districts.

#### Interaction challenges

The participants mentioned challenges related to teamwork and the transmission of information as contributors to human errors. For example, working in pairs was mentioned as a risk factor. If work partners do not get along well, cooperation may not be as high in quality as it should be. On the other hand, if work partners get along too well, and are too familiar with each other, some matters or essential procedures may be forgotten.

*If you have a good*,* familiar working partner and you know the job goes well*,* you just forget in a situation*,* or you just don’t do something because [you think]*,* “We don’t make errors because we work so well together.” (P3)*.

According to the participants, if one paramedic is at an advanced level and the other one is at a basic level, the paramedic with the more education may dominate decision-making and not acknowledge the basic-level paramedic. In addition, if a paramedic is inexperienced, it can cause them to feel uncertainty and lack the courage to voice their thoughts about a situation to a more experienced paramedic, which creates a risk for human error. Inexperience may also cause challenges if the partners do not trust each other or if one partner doubts their less-experienced partner’s decision-making. Conversely, there is also a risk of error if an experienced paramedic is overly trusting of the competence of a student who is doing their practical training and gives them too much responsibility.

*[There can be] a strong-willed*,* experienced advanced-level paramedic and then there is a basic level*,* fairly inexperienced paramedic who hasn’t been listened to during this shift*,* whose lead hasn’t listened to them at all. Will they be heard in that moment when it would actually be reasonable if the other one is irritated*,* tired*,* and just moves on? (P6)*

The importance of communication when working in pairs was emphasized in the interviews. Insufficient communication can decrease situational awareness, cause a breakdown in communication, and increase misunderstandings. Unclear communication or situations in which one of the working partners does not maintain situational awareness or participate in decision-making may lead to information not being shared. In such cases, one paramedic must work alone and make decisions by themselves.

*You are trying to say something*,* but your working partner doesn’t understand for one reason or another; in other words*,* communication is unclear or incomplete*,* or you don’t know where you are going [in the situation]. In our minds*,* we may be treating two different patients. (P7)*

#### Challenges related to technological systems

According to the participants, paramedics can be overly trusting of technological systems, and they often make assumptions without checking the accuracy of the information. Rarely used devices may cause human error if something goes unnoticed while a treatment is being performed, especially if a long time has passed since the previous training session.

*You have had training*,* but you don’t remember how to use this device anymore. A device that is rarely used*,* for instance*,* external pacing; if you forget something*,* some nuance gets overlooked. (P12)*

### Paramedics themselves

**Fig. 4:**
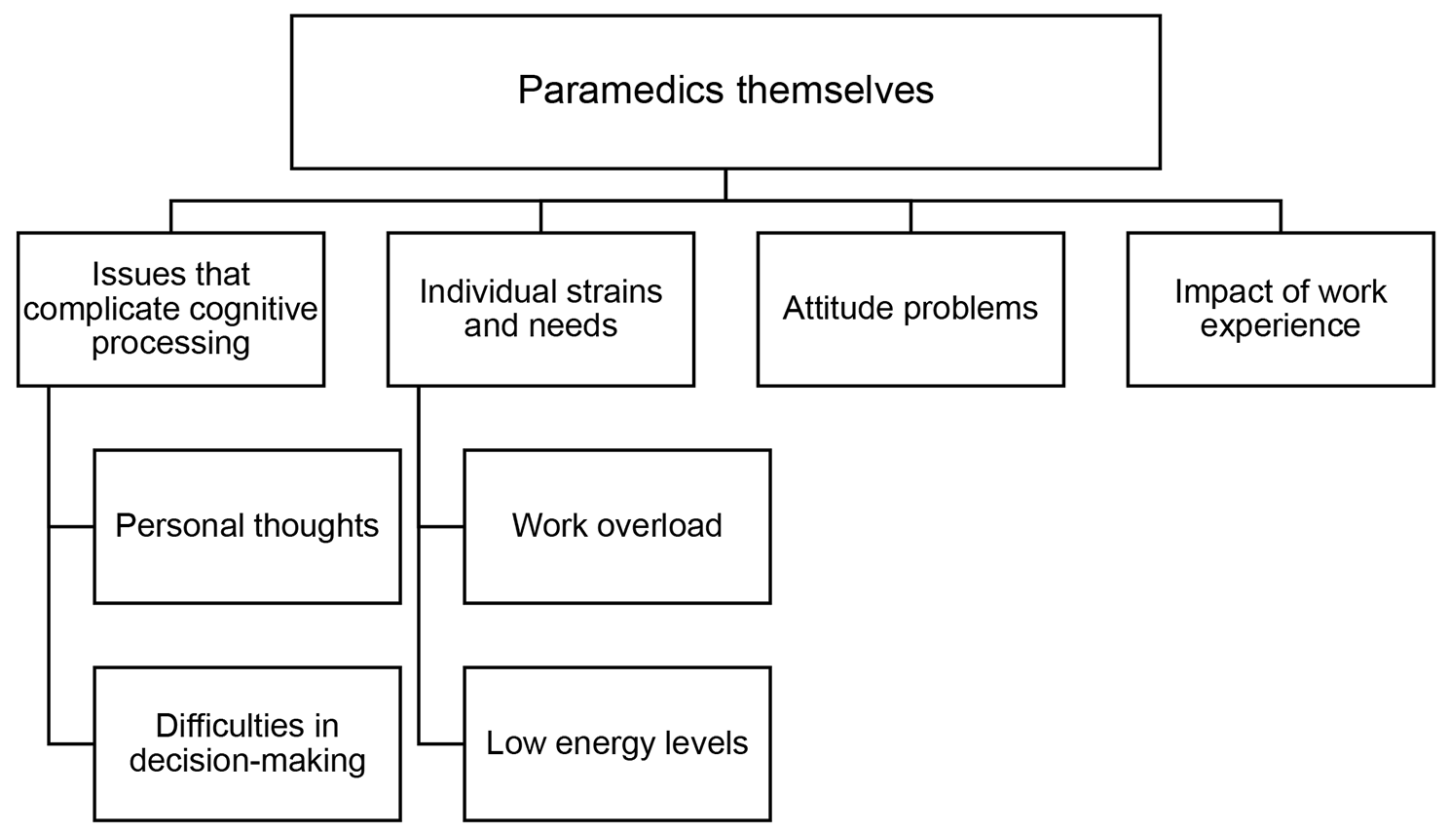
The third main category, *Paramedics themselves*

The main category *Paramedics themselves* consisted of four generic categories: *Issues that complicate cognitive processing*, *Individual strains and needs*, *Attitude problems* and *Impact of work experience*. These categories consist of aspects that are mostly linked to paramedics’ personal issues. Figure 4 provides an overview of the third main category.

#### Issues that complicate cognitive processing

***Personal thoughts.*** Participants mentioned that a paramedic with emotionally stressful personal issues may have decreased concentration at work. When their thoughts are somewhere else, a paramedic may do something that is not necessary or that should be done in some other way. The first work shift after vacation was also mentioned as a factor that contribute to human error.

*There is something else stressing you out, in your personal life. (P9)*

***Difficulties in decision-making.*** According to the participants, high-pressure situations in which paramedics must make quick decisions, weighing the pros and cons thereof, can contribute to human error. Errors can happen when a decision must be made but not all the essential issues of the situation are acknowledged. It was mentioned that fast information processing and information overflow can contribute to errors. A decision to not convey a patient to the hospital after evaluating their condition was mentioned as an example of a human error that may occur in such situations.

*The errors happen when the decisions have to be made—you make*,* for example*,* a wrong decision or… I don’t want to say “the wrong decision” but you don’t acknowledge everything possible in your decision-making. (P7)*

#### Individual strains and needs

***Work overload.*** Work overload was mentioned as a factor contributing to human error because it can cause a lapse in concentration. Even if a paramedic has sufficient competence and knowledge, the accumulation of stressful situational factors may lead to errors. Furthermore, young paramedics may find it difficult to accept that errors occur and be afraid of making them, which increases their stress during their spare time as well.

*There is pressure in this role*,* and it feels uncomfortable to be under pressure*,* and even after five years of work experience*,* I still feel uncomfortable. Working under pressure is not nice*,* but you have to try to bear it (P5)*

***Low energy levels.*** According to the participants, energy levels are lower at night, which increases the risk of human error. Specifically, early hours were mentioned as a time period that has a higher risk of errors. Fatigue may also be a contributing factor, for instance, if there are many missions during one shift. According to the participants, fatigue can lead to errors because something important may not be noticed or asked about during a patient’s assessment. Tiredness can lessen one’s ability to concentrate, leading to a risk of misunderstanding, and the conceptualization of the overall situation may be distorted. In addition, when paramedics are tired during their shift, they may ask for a doctor to order non-conveyance of a patient without a sufficient interview or examination. Fatigue can weaken the decision-making process as a whole.

*You are tired*,* tired and hungry and you decide a little bit too quickly*,* for example*,* to not take a patient to the hospital by ambulance. With that rapid phone call to a doctor*,* you make a decision*,* and then perhaps there ends up being an issue that is not noticed. (P12)*

Hunger was mentioned as contributing to human error; it may cause the paramedics to speed up, either unconsciously or intentionally, managing a mission quickly when they want to eat. Hunger was claimed to be a simple and unambiguous cause of errors in EMS settings.

*It doesn’t need anything other than hunger, hurry, and fatigue. (P14)*

#### Attitude problems

Low motivation toward work is strongly related to human error; it can lessen one’s understanding of the seriousness of a situation and negatively affect professional skills. The participants explained that if a paramedic is not highly motivated, leaving for a mission can be annoying, and a negative attitude can also affect colleagues’ dynamics. Learned working models, a negative attitude towards new information, and a stubbornness to do things one’s own way can also contribute to human error.

*If the attitude is “could not care less*,*” and when motivation eats your own professional skills*,* kind of…even though you are a skilled professional*,* if you don’t have the right attitude in that situation. (P5)*

#### Impact of work experience

The participants mentioned that both inexperienced and a very long experience can cause human error, although for different reasons. The participants felt that an inexperienced working partner may not have enough competence in a situation, which can cause errors to happen. A paramedic not having experience in a certain mission type can increase stress levels and even manifest in incapacitation or enacting the wrong procedure. Inexperience can also cause an inability to reflect one’s competence or one believing that one’s skills are limited. Furthermore, the participants mentioned that an inexperienced paramedic may feel the need to show off or be afraid of admitting their ignorance, especially if they only have a fixed-term job contract. Keeping a lack of competence hidden may lead to errors.

*If*,* for example*,* there are two inexperienced paramedics working together*,* human error can happen because they are not quite sure what they are doing. (P15)*

However, the participants argued that long experience can also be a risk factor for human errors. Long experience can cause indifference and adherence to one’s working manners. A very experienced paramedic might do things automatically and not stop to think about what they are doing, and some patient safety procedures may be forgotten.

*An experienced [paramedic]*,* they do things out of instinct*,* they don’t stop to think about those issues*,* and they don’t check everything. (P11)*

## Discussion

In this study, our aim was to investigate paramedics’ opinions on the factors contributing to human errors. In the analysis, the main categories that were identified were *Changing work environment*,* Organization of work*, and *Paramedics themselves*. The analysis showed the interaction between the system and the paramedics, and how paramedics should be able to adapt their performance in different circumstances. These results support the prevalent theory of human error [4].

There are many factors related to working in the EMS that can contribute to human error. The findings of this study showed that paramedics must adapt to challenging and dynamic environments and circumstances. These situations may contribute to human error. In accordance with previous studies, a sense of urgency is a considerable stress factor in EMS settings [8, 21–24]. Our results found that challenging work conditions can affect paramedics’ work, which is in line with the study by Bigham et al. [12]. Other emergency service professionals such as firefighters and first responders may face similar challenges as they work in the same prehospital emergency care setting, however, future studies are needed about the differences of challenges between these professions.

Relying too much on mission codes indicates that cognitive biases are common in the EMS [25]. If paramedics are overly reliant on dispatch information, they may have preconceived assumptions about a patient’s condition, which can cause bias [26], and contribute to human error. In addition, emergency response driving includes many risks of errors in EMS settings, also found in a previous study [27].

Particular patient groups can change work circumstances and require performance variability. In that way, those situations may contribute to errors. Treating critically ill patients in EMS settings includes many stressors that can affect paramedics’ work [23, 28]. Another patient group that was mentioned was frequent callers. This study indicated that there may be a risk of ignoring necessary information with frequent callers or anchor information that is easily available with critically ill patients and as a result, the vigilance to notice other possible factors that may affect the condition could be reduced [25]. However, this phenomenon would require more focused studies.

Inadequate care guidelines may also contribute to human error; these findings indicate that many contributing factors are system-level issues which support the system approach to human error [3]. Moreover, previous studies have demonstrated that a lack of SOPs increases intuitive thinking processes that can expose individuals to cognitive biases [29–31]. A study by Diller et al. [29] showed that communication problems can stop the flow of information or cause misunderstandings. This study adds that there are many aspects and challenges related to teamwork that can negatively impact patient safety and care in EMS.

Paramedics must process large amounts of information in dynamic environments during EMS missions [32], and unique and multidimensional decision-making can create a risk for patient safety [33, 34]. Factors related to the work environment, such as unsafe scenes and time pressure, can challenge paramedics’ decision-making [24, 34]. Decision-making support systems might be a way for EMS organizations to support paramedics in their work and improve patient safety, for instance, using electronic SOPs as a decision supporting tool can improve patient safety [35]. However, further studies are needed about the factors that affect paramedics’ decision-making, and how paramedics decision-making can be supported under stressful circumstances.

The findings regarding work-related stress support evidence from previous studies [6, 12]. Personal issues that are emotionally stressful can negatively impact paramedics’ concentration and patient safety. Many studies have investigated fatigue in the EMS setting, and the findings of this study are consistent therewith; fatigue creates risks related to both patient and occupational safety [6, 7, 24, 36]. However, this study provides insight into how fatigue affects EMS workers from paramedics’ perspectives.

Our findings showed that attitude problems can contribute to human error. Many factors, including occupational stress, can reduce paramedics’ motivation to work. A few studies have indicated that EMS-specific factors, such as stressful and challenging environments, as well as occupational factors, can cause job dissatisfaction and negatively impact patient safety [37, 38]. This is one of many reasons why organizations should become aware of these system-level issues and support paramedics’ psychological well-being at work. Moreover, further studies should examine, for instance, work motivation, and organizational factors.

Professional competence plays a key role in managing missions with different challenges in the EMS, which is why support from colleagues is needed [39]. The results of this study suggested that very experienced paramedics have set routines, which can create a risk for error. In addition, routine matters may not need particular attention, but external factors or interruptions can cause errors to occur [3, 29]. The dynamic and complex work environment of the EMS can create favorable circumstances for routine-based errors if organizations do not understand and prepare for these factors.

### Methodological considerations

Potential participants were recruited via social media with the aim of getting a wide representation from across Finland, and paramedics working in different parts of Finland could be best reached through a specific social media group. Using a social media platform for participant recruitment has limitations, as only those who use this platform could be reached, which could affect the number and the homogeneity of potential participants [40]. Still, more traditional recruitment methods have similar challenges and recruiting participants through social media has been found to be a useful and valid method [41]. All of the volunteer paramedics who were interested were included in this study. Their own interest can be assessed as at least partly stemming from the perception that they had a lot to contribute to the research. This supported the common goal of qualitative research of achieving in-depth understanding of the studied topic [42]. The data was saturated, meaning that no additional aspects were mentioned [43] and in-depth results can be assessed as achieved. Moreover, although qualitative research does not aim for broad generalizations [42], the convenience sampling method could limit the transferability of the results [40].

The inclusion criteria were that paramedics should be basic- or advanced-level paramedics or EMS field supervisors with any length of work experience and who worked in EMS at the time of recruitment. No other characteristics of the participants, such as age or education, were collected beyond gender, occupation, and EMS area. This is because in the recruitment letter, the potential participants were assured that their personal information would not be recorded. A lack of background information can be considered either a strength or a limitation. Still, during the interviews, all the participants share that they had several years of work experience in the EMS, and they represented various EMS areas in Finland. Selecting participants with different lengths of work experience could have produced more varied insights into the topic; however, there was a limited number of interested paramedics, hence any purposive sampling could not be used.

The first author conducted the interviews by herself. Clinical experience in the EMS setting was beneficial for asking specific follow-up questions during the interviews and gaining a deeper understanding of the participants’ perspectives. However, the interviewer’s pre-understanding of the research topic may have caused some bias toward the subject. During the interviews, the participants were encouraged to outline factors contributing to human errors by describing situations in which they had made an error or “a near miss” situation. However, that was voluntary, and the participants were not pressured to talk about such situations if they were a sensitive topic for them.

Interviews were conducted in three different ways (face-to-face, online and by phone) which can be seen both a strength or a limitation. With the use of multiple data collection methods, more participants could be reached because different methods allow access to geographically wider areas and a participant could choose the most appropriate method for themselves [44–46]. However, there may be challenges to build a rapport between the participant and the interviewer, for instance, in phone interviews. Moreover, the quality and depth of research data can vary when using multiple data collection methods [45]. Considering the aim and sampling of this study, multiple data collection methods were seen as appropriate for capturing an in-depth view of the research topic.

## Conclusions

The paramedics recognized various factors that can contribute to human error in the EMS setting. Although the findings revealed that many of the contributing factors related to the paramedics themselves, system-level matters were also found to affect paramedics’ work and paramedics must adapt to different circumstances. Our findings shed new light on research in this area by investigating human error proactively from paramedics’ point of view. However, further qualitative and quantitative research is needed to form a deeper understanding of contributing factors of human error in the EMS setting.

### Recommendations for future practice

To understand contributors to human errors at the level of practice and proactively, many individual and system-level matters should be acknowledged. Organizations and educational institutions can use the findings of this study to develop and refine procedures and supporting systems for paramedics, thereby improving patient safety.

### Electronic supplementary material

Below is the link to the electronic supplementary material.

Supplementary Material 1

Supplementary Material 2

Supplementary Material 3

Supplementary Material 4

## Data Availability

The datasets generated and analyzed during the current study are not publicly available for ethical reasons. The informed consent contained a statement that only researchers have access to the raw data and the findings would be presented in an anonymized way.

## Abbreviations

EMS: Emergency medical services
COREQ: Consolidated criteria for reporting qualitative research
UAS: University of Applied Sciences
SOP: Standard operating procedure

## References

[1] Liberati EG, Peerally MF, Dixon-Woods M. Learning from high risk industries may not be straightforward: a qualitative study of the hierarchy of risk controls approach in healthcare. Int J Qual Health Care. 2018;30(1):39–43. 29300992 10.1093/intqhc/mzx163 PMC5890869

[2] Ruth CK. Human factors contributing to nursing errors [Dissertation]: University of Texas at Tyler; 2014.

[3] Hollnagel E, Safety-I. and Safety-II. The past and future of safety management. 1st ed. Boca Raton: CRC Press Taylor & Francis Group; 2014.

[4] Read GJM, Shorrock S, Walker GH, Salmon PM. State of science: evolving perspectives on ‘human error’. Ergonomics. 2021;64(9):1091–114. 34243698 10.1080/00140139.2021.1953615

[5] Roth C, Brewer M, Wieck KL. Using a Delphi Method to identify human factors contributing to nursing errors. Nurs Forum. 2017;52(3):173–9. 27434130 10.1111/nuf.12178

[6] Patterson PD, Weaver MD, Frank RC, Warner CW, Martin-Gill C, Guyette FX, et al. Association between poor sleep, fatigue, and safety outcomes in emergency medical services providers. Prehosp Emerg Care. 2012;16(1):86–97. 22023164 10.3109/10903127.2011.616261 PMC3228875

[7] Donnelly EA, Bradford P, Davis M, Hedges C, Socha D, Morassutti P. Fatigue and safety in Paramedicine. CJEM. 2019;21(6):762–5. 31771693 10.1017/cem.2019.380

[8] Hagiwara MA, Magnusson C, Herlitz J, Seffel E, Axelsson C, Munters M, et al. Adverse events in prehospital emergency care: a trigger tool study. BMC Emerg Med. 2019;19(1):14. 30678636 10.1186/s12873-019-0228-3 PMC6345067

[9] Andersson U, Maurin Söderholm H, Wireklint Sundström B, Andersson Hagiwara M, Andersson H. Clinical reasoning in the emergency medical services: an integrative review. Scand J Trauma Resusc Emerg Med. 2019;27(1):76. 31426839 10.1186/s13049-019-0646-y PMC6700770

[10] Wasserberger J, Ordog GJ, Donoghue G, Balasubramaniam S. Base station prehospital care: judgement errors and deviations from protocol. Ann Emerg Med. 1987;16(8):867–71. 3619166 10.1016/S0196-0644(87)80524-3

[11] Crossman M. Technical and Environmental Impact on Medication Error in Paramedic Practice: a review of causes, consequences and strategies for Prevention. J Emerg Prim Health Care. 2009;7(3).

[12] Bigham BL, Buick JE, Brooks SC, Morrison M, Shojania KG, Morrison LJ. Patient safety in emergency medical services: a systematic review of the literature. Prehosp Emerg Care. 2012;16(1):20–35. 22128905 10.3109/10903127.2011.621045

[13] Misasi P, Keebler JR. Medication safety in emergency medical services: approaching an evidence-based method of verification to reduce errors. Ther Adv Drug Saf. 2019;10:2042098618821916. 30728945 10.1177/2042098618821916 PMC6351968

[14] Dicicco-Bloom B, Crabtree BF. The qualitative research interview. Med Educ. 2006;40(4):314–21. 16573666 10.1111/j.1365-2929.2006.02418.x

[15] Tong A, Sainsbury P, Craig J. Consolidated criteria for reporting qualitative research (COREQ): a 32-item checklist for interviews and focus groups. Int J Qual Health Care. 2007;19(6):349–57. 17872937 10.1093/intqhc/mzm042

[16] Health Care Act. Stat. 1326 (2010).

[17] Degree of prehospital emergency care. Stat 585 (2017).

[18] Raatiniemi L, Brattebo G. The challenge of ambulance missions to patients not in need of emergency medical care. Acta Anaesthesiol Scand. 2018;62(5):584–7. 29520763 10.1111/aas.13103

[19] Stratton SJ. Population Research: convenience sampling strategies. Prehosp Disaster Med. 2021;36(4):373–4. 34284835 10.1017/S1049023X21000649

[20] Elo S, Kyngäs H. The qualitative content analysis process. J Adv Nurs. 2008;62(1):107–15. 18352969 10.1111/j.1365-2648.2007.04569.x

[21] Cushman JT, Fairbanks RJ, O’Gara KG, Crittenden CN, Pennington EC, Wilson MA, et al. Ambulance personnel perceptions of near misses and adverse events in pediatric patients. Prehosp Emerg Care. 2010;14(4):477–84. 20662679 10.3109/10903127.2010.497901 PMC2932803

[22] Edland A, Svenson O. Judgment and decision making under time pressure. In: Svenson O, Maule AJ, editors. Time pressure and stress in Human Judgment and decision making. Boston (MA): Springer; 1993.

[23] Lammers R, Byrwa M, Fales W. Root causes of errors in a simulated prehospital pediatric emergency. Acad Emerg Med. 2012;19(1):37–47. 22251191 10.1111/j.1553-2712.2011.01252.x

[24] Galy E, Cariou M, Mélan C. What is the relationship between mental workload factors and cognitive load types? Int J Psychophysiol. 2012;83(3):269–75. 22008523 10.1016/j.ijpsycho.2011.09.023

[25] Graber ML, Franklin N, Gordon R. Diagnostic error in internal medicine. Arch Intern Med. 2005;165(13):1493–9. 16009864 10.1001/archinte.165.13.1493

[26] Johansson H, Lundgren K, Hagiwara MA. Reasons for bias in ambulance clinicians’ assessments of non-conveyed patients: a mixed-methods study. BMC Emerg Med. 2022;22(1). 10.1186/s12873-022-00630-8 PMC9074185 35524195

[27] Jakonen A, Manty M, Nordquist H. Safety checklists for Emergency Response Driving and Patient Transport: experiences from Emergency Medical services. Jt Comm J Qual Patient Saf. 2021;47(9):572–80. 34183282 10.1016/j.jcjq.2021.05.008

[28] Lauria MJ, Gallo IA, Rush S, Brooks J, Spiegel R, Weingart SD. Psychological skills to improve Emergency Care Providers’ performance under stress. Ann Emerg Med. 2017;70(6):884–90. 28460863 10.1016/j.annemergmed.2017.03.018

[29] Diller T, Helmrich G, Dunning S, Cox S, Buchanan A, Shappell S. The human factors analysis classification system (HFACS) applied to health care. Am J Med Qual. 2014;29(3):181–90. 23814026 10.1177/1062860613491623

[30] Saposnik G, Redelmeier D, Ruff CC, Tobler PN. Cognitive biases associated with medical decisions: a systematic review. BMC Med Inf Decis Mak. 2016;16(1):138. 10.1186/s12911-016-0377-1 PMC5093937 27809908

[31] Pedersen I, Solevåg AL, Trygg Solberg M. Simulation-based training promotes higher levels of cognitive control in acute and unforeseen situations. Clin Simul Nurs. 2019;34:6–15. 10.1016/j.ecns.2019.05.003

[32] Sedlár M. Cognitive skills of emergency medical services crew members: a literature review. BMC Emerg Med. 2020;20(1):44. 32471352 10.1186/s12873-020-00330-1 PMC7257132

[33] Reay G, Rankin JA, Smith-MacDonald L, Lazarenko GC. Creative adapting in a fluid environment: an explanatory model of paramedic decision making in the pre-hospital setting. BMC Emerg Med. 2018;18(1):42. 30442096 10.1186/s12873-018-0194-1 PMC6238402

[34] Bijani M, Abedi S, Karimi S, Tehranineshat B. Major challenges and barriers in clinical decision-making as perceived by emergency medical services personnel: a qualitative content analysis. BMC Emerg Med. 2021;21(1):1–12. 33468045 10.1186/s12873-021-00408-4 PMC7815282

[35] Bashiri A, Alizadeh Savareh B, Ghazisaeedi M. Promotion of prehospital emergency care through clinical decision support systems: opportunities and challenges. Clin Exp Emerg Med. 2019;6(4):288–96. 31910499 10.15441/ceem.18.032 PMC6952626

[36] Ramey S, MacQuarrie A, Cochrane A, McCann I, Johnston CW, Batt AM. Drowsy and dangerous? Fatigue in paramedics: an overview. Ir J Paramedicine. 2019;4(1):1–9.

[37] Eiche C, Birkholz T, Konrad F, Golditz T, Keunecke JG, Prottengeier J. Job satisfaction and performance orientation of paramedics in German Emergency Medical Services-A Nationwide Survey. Int J Environ Res Public Health. 2021;18(23). 10.3390/ijerph182312459 PMC8656590 34886189

[38] Hammer JS, Mathews JJ, Lyons JS, Johnson NJ. Occupational stress within the paramedic profession: an initial report of stress levels compared to hospital employees. Ann Emerg Med. 1986;15(5):536–9. 3963532 10.1016/S0196-0644(86)80988-X

[39] Hörberg A, Jirwe M, Kalén S, Vicente V, Lindström V. We need support! A Delphi study about desirable support during the first year in the emergency medical service. Scand J Trauma Resusc Emerg Med. 2017;25(1):89. 28877728 10.1186/s13049-017-0434-5 PMC5588605

[40] Gill SL. Qualitative sampling methods. J Hum Lactation. 2020;36(4):579–81. 10.1177/0890334420949218 32813616

[41] Thornton L, Batterham PJ, Fassnacht DB, Kay-Lambkin F, Calear AL, Hunt S. Recruiting for health, medical or psychosocial research using Facebook: systematic review. Internet Interventions. 2016;4:72–81. 30135792 10.1016/j.invent.2016.02.001 PMC6096238

[42] Polit DF, Beck CT. Generalization in quantitative and qualitative research: myths and strategies. Int J Nurs Stud. 2010;47(11):1451–8. 20598692 10.1016/j.ijnurstu.2010.06.004

[43] Mwita K. Factors influencing data saturation in qualitative studies. Int J Res Bus Social Sci. 2022;11(4):2147–4478.

[44] Heath J, Williamson H, Williams L, Harcourt D. It’s just more personal: using multiple methods of qualitative data collection to facilitate participation in research focusing on sensitive subjects. Appl Nurs Res. 2018;43:30–5. 30220360 10.1016/j.apnr.2018.06.015

[45] Deakin H, Wakefield K. Skype interviewing: reflections of two PhD researchers. Qualitative Res. 2013;14(5):603–16. 10.1177/1468794113488126

[46] Opdenakker R. Advantages and disadvantages of four interview techniques in qualitative research. Forum: Qualitative Social Res / Qualitative Sozialforschung. 2006;7(4):1.

