# Supplementary material for: Human errors in emergency medical services: a qualitative analysis of contributing factors
Source: Scand J Trauma Resusc Emerg Med. 2024 Aug 30;32:78. doi: 10.1186/s13049-024-01253-7 (PMC11363522; doi:10.1186/s13049-024-01253-7)
Supplement: Supplementary file 2 — Supplementary Material 2 [file 13049_2024_1253_MOESM2_ESM.docx]

Additional file 2. The interview schedule.

1. What does human error mean to you?
   1. Follow-up question(s) if needed.
2. Can you describe situations where human error can happen in an EMS setting?
   1. Follow-up question(s) if needed; can you describe this in more detail?
   2. What kind of issues affect the situation you described?
3. Can you describe actions where human error can happen in an EMS setting?
   1. Follow-up question(s) if needed; can you describe this in more detail?
