# Supplementary material for: Human errors in emergency medical services: a qualitative analysis of contributing factors
Source: Scand J Trauma Resusc Emerg Med. 2024 Aug 30;32:78. doi: 10.1186/s13049-024-01253-7 (PMC11363522; doi:10.1186/s13049-024-01253-7)
Supplement: Supplementary file 3 — Supplementary Material 3 [file 13049_2024_1253_MOESM3_ESM.docx]

Additional file 3. Examples of how the categories were formulated.

| Representative quotations related to the study’s aim | Heading | Sub-categories | Generic categories | Main category |
| --- | --- | --- | --- | --- |
| *“There is a rushed situation, and you are like, “Okay, it is this,” and then you give the wrong dose [of medication to the patient] — that rushed situation has created a pressure in a way, and that is why an error happens.”* (P12) | A rushed situation adds pressure to work in a way that error can happen. (P12) | Urgency | The nature of the work | Changing work environment |
| *"The feeling of being rushed, if, for example, deadlines are given for missions - time pressure arises from them, and then it may change one's behavior and the way the task is carried out, and something may be left undone due to the rush."* (P7) | Urgency and time pressure change paramedics’ behavior or the way the task is carried out and then something may be left undone. (P7) |  |  |  |
| *“Of course, you must wear ear buds all the time and you have to listen to certain channels, and if you are focusing on something…it will disturb your own work.”* (P3) | Ear buds and communication using them cause a lapse of concentration and this will disturb one’s work. (P3) | Disruptive, external issues |  |  |
| *“Then you don't pay attention to everything, like “You are listening to ear bud, you're not listening to the patient.””* (P8) | Radio communication causes a lapse of concentration and disturb paramedics’ work. (P8) |  |  |  |
| *“We had a patient with an epileptic seizure the previous day, and I had given them an intranasal medication. Without thinking, I gave the same dose of medication intravenously to the next patient, like I had a day before.”* (P15) | A similar patient in previous days but the current patient needs a bit different care. (P15) | Particular patient groups | Factors related to missions |  |
| *"But then the downside is that when you see that it's one of those again, like a regular customer who always has the same issue and usually there's nothing wrong, it may affect your attitude towards the situation... This kind of situation, we could perhaps talk about being burn out, where you see the same thing over and over again for the same reason, even though the situation might be slightly different."* (P7) | Even if the situation might be slightly different, previous visits and a frequent caller can make paramedics frustrated, and the attitude towards the situation is wrong. (P7) |  |  |  |
| *“That disregard in terms of particular missions and patient groups—you don’t have enough strength with these same situations. We visit them 10 times for the same reason, and one day there is a real symptom, but you don’t have an interest in this patient anymore and when there is a real symptom, paramedics may ignore that because of the patient’s background.”* (P8) | Paramedics may ignore the patient’s symptoms if the patient is a frequent caller, and situations are often the same. (P8) |  |  |  |
