# Supplementary material for: Human errors in emergency medical services: a qualitative analysis of contributing factors
Source: Scand J Trauma Resusc Emerg Med. 2024 Aug 30;32:78. doi: 10.1186/s13049-024-01253-7 (PMC11363522; doi:10.1186/s13049-024-01253-7)
Supplement: Supplementary file 4 — Supplementary Material 4 [file 13049_2024_1253_MOESM4_ESM.docx]

Additional file 4. Factors contributing to errors, according to paramedics.
